# 18F–FDG PET/CT scan confirmed by pathology findings in a singular case of squamous cell carcinoma of the epiglottis

**DOI:** 10.1186/s41824-017-0012-0

**Published:** 2017-11-15

**Authors:** G. M. Lima, A. Matti, L. Burgio, C. Nanni, P. Castellucci, S. Fanti

**Affiliations:** 1 Nuclear Medicine Department, S. Orsola-Malpighi Hospital, University of Bologna, Bologna, Italy; 2 ENT Department, S. Orsola-Malpighi Hospital, University of Bologna, Bologna, Italy

## Abstract

**Background:**

Only about 1% of all head and neck lateral or paramedian cancers described in the scientific literature shows, in staging, contralateral cervical adenopathy without ipsilateral pathological involvement of lymph nodes.

**Case Presentation:**

This case is one of them, in which 18F–FDG PET/CT scan is confirmed by pathology findings, and has correctly identified all metastatic disease foci.

**Conclusions:**

To date, PET/CT is not recommended in head and neck cancer staging. However, the use of PET/CT in head and neck cancer staging can define possible metastatic disease foci, clarify c.e. CT suspicious findings and, in some cases, change the TNM stage, with a strong prognostic and therapeutic impact.

## Background

Only about 1% of all head and neck lateral or paramedian cancers described in the scientific literature shows, in staging, contralateral cervical adenopathy without ipsilateral pathological involvement of lymph nodes.

## Case Presentation

We present a case of a 55 years old man with a lateral cervical left mass, and an expansive lesion in lingual epiglottis versant, on the right side (involving ipsilateral epiglottic vallecula).

The patient underwent staging procedures including contrast enhancement CT (c.e.CT) of the head and neck region and a whole body [18F]-FDG PET/CT. Accurate pre-treatment staging of cervical lymph nodes is essential for proper treatment planning. Moreover, regional lymph nodes involvement is one of the utmost prognostic factors regarding the patient’s outcome. To date, there is still no consensus on the best imaging modality that should be used in head and neck cancer staging, between CT, MRI and [18F]-FDG PET/CT (Kastrinidis et al., 2013).

**Fig. 1:**
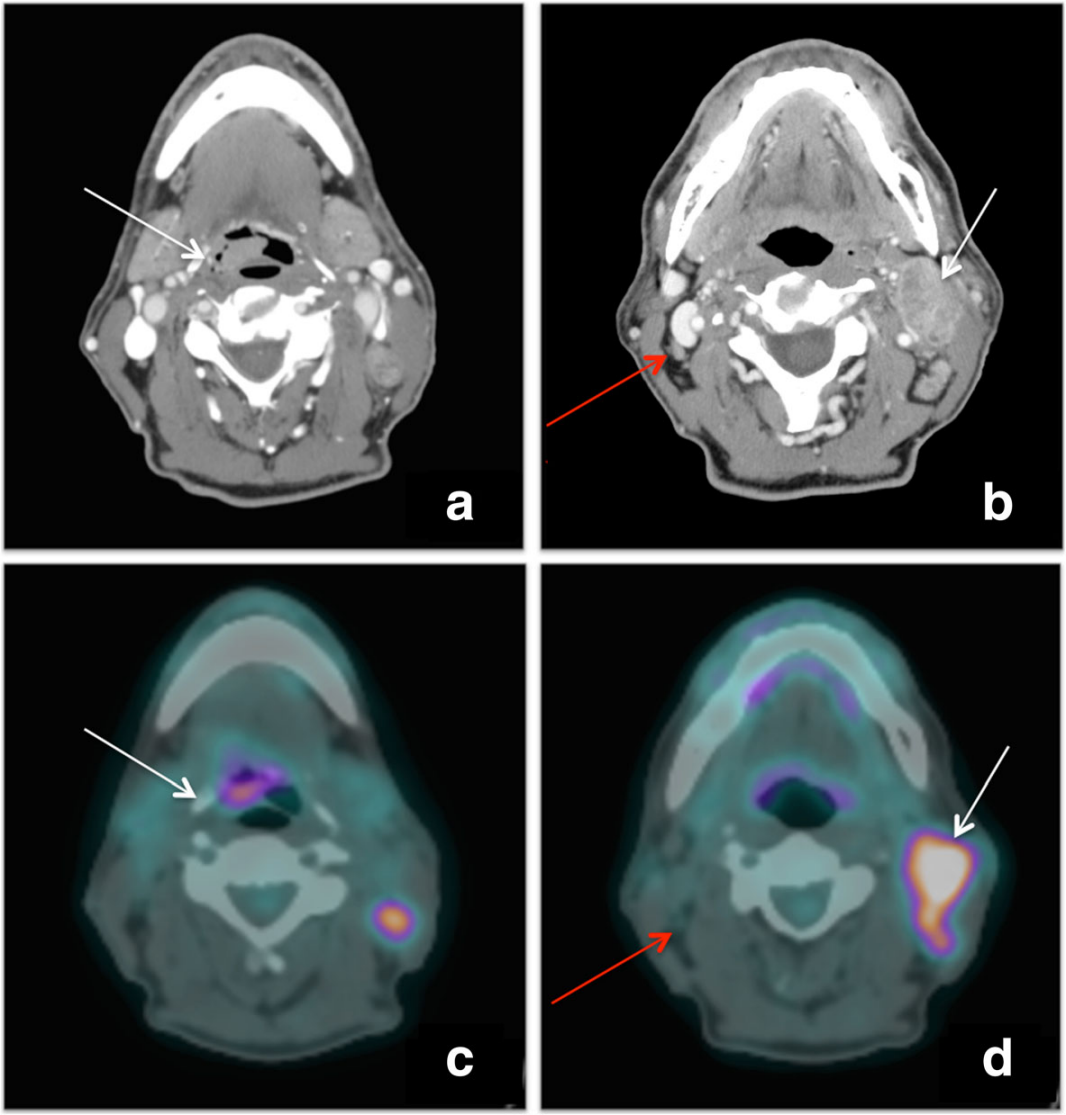
**a** transaxial c.e.CT: primary epiglottic lesion. **b** transaxial c.e.CT: lateral cervical lymph nodes. **c** transaxial PET/CT: hypermetabolic primary epiglottic lesion. **d** transaxial PET/CT: not pathological right lateral cervical lymph nodes, left hypermetabolic lateral cervical adenopathy

The first staging examination, a c.e.CT, showed a neck paramedian right lesion presenting an intense contrast enhancement (measuring 18 × 8 mm), contralateral confluent lymph nodes compressing the jugular vein and ipsilateral small lymph nodes, of unclear nature. Figure 1-a: transaxial c.e.CT scan shows primary epiglottic lesion (white arrow); Fig. 1-b: transaxial c.e.CT scan shows both the increased left lateral cervical lymph nodes (white arrow), both some small right lateral cervical lymph nodes with contrast enhancement (red arrow).

Overall, in the radiological report, c.e.CT was doubtful for ipsilateral adenopathy.

Staging continued with a whole body [18F]-FDG PET/CT to assess possible metastatic disease foci and to define the nature of the ipsilateral cervical lymph-nodes.

A whole-body PET scan was acquired, 3 min per bed position, one hour after the injection of a standard activity (3,5 MBq/Kg) of 18F–FDG. It was also performed a low-dose CT for the attenuation correction (120 kV, 80 mA, 0.8 s per rotation, thickness 3,75 mm).

PET scan showed an increased FDG uptake of the primary lesion in the right epiglottis (SUVmax = 10, SUVmean = 6.3, MTV = 1.9 cm3, TLG = 12) and also in correspondence of the II and III level in the contralateral side (SUVmax = 15, SUVmean = 8.5, MTV = 14.9 cm3, TLG = 126.6). Figure 1-c: transaxial PET/CT scan shows an increased [18F]-FDG uptake area on the right versant of the epiglottis (white arrow); Fig. 1-d: transaxial PET/CT scan demonstrates clearly that right lateral cervical lymph nodes are not pathological (red arrow) and confirm, on the left side, the adenopathy (white arrow).

Noticeably, [18F]-FDG PET/CT showed no significant FDG uptake areas in the ipsilateral right cervical chain. The minimal activity of the right-sided lymph node was indistinguishable from the background activity (SUVmax = 1.5), suggesting its benign nature (Lim et al., 2016).

After staging (T1-N2b), patient underwent surgical supraglottic laryngectomy and bilateral lateral cervical lymph nodes dissection.

Histopathology results showed a squamous cell carcinoma of the right lingual epiglottis side, no malignant cells in the right lateral cervical lymph nodes, while metastatic cells in the left lateral cervical lymph nodes were detected (pT1-N2b).

## Discussion

[18F]-FDG PET/CT report was confirmed by pathology, identifying the primary epiglottic carcinoma and addressing as metastatic just the contralateral lymph nodes.

[18F]-FDG PET/CT properly assessed as negative the ipsilateral lymph nodes that were suspected to be metastatic at c.e.CT.

The presence of the contralateral lymph node metastasis without ipsilateral lesion, is a very rare event since it may occur in about 1% of all head and neck cancers (Rucci et al., 1990); in this case, the primary mass on the right lingual epiglottis side was very close to midline, so we could have expected, at least, a bilateral cervical involvement, but not a solitary contralateral adenopathy.

[18F]-FDG PET/CT scan confirmed, in this patient, to be an accurate method to stage head and neck cancer.

However, to date there is not a single staging modality that could spare these patients a bilateral neck dissection. Patients could also benefit from a sentinel node investigation in order to identify the primary lymphatic way and, if the lymphatic drainage side is same as the pathological nodes identified by PET, only contralateral dissection may be considered.

At this time, [18F]-FDG PET/CT is considered an important imaging modality in the evaluation of squamous cell carcinoma of the head and neck (Quon et al., 2007), especially for the detection of regional nodal metastasis (Sun et al., 2015; Schöder & Yeung, 2004).

However, the indications to PET scan, in head and neck cancer staging, are not completely exploited, compared to its application in restaging, assessment after-surgery, radiotherapy and follow-up.

## Conclusion

Whole body [18F]-FDG PET/CT, used in staging, can identify metastatic foci, clarify c.e.CT suspicious findings and, in some cases, change the TNM stage, with a strong prognostic and therapeutic impact.
